# What is the failure rate in extending labour analgesia in patients with a body mass index ≥ 40 kg/m^2^compared with patients with a body mass index < 30 kg/m^2^? a retrospective pilot study

**DOI:** 10.1186/s12871-015-0095-8

**Published:** 2015-08-01

**Authors:** Victoria A. Eley, Andre van Zundert, Leonie Callaway

**Affiliations:** 1 Department of Anaesthesia and Perioperative Medicine, Royal Brisbane and Women’s Hospital, Butterfield St, Herston, 4006 Queensland, Australia; 2 School of Medicine, The University of Queensland, Herston Rd, Herston, 4006 Queensland, Australia; 3 Department of Internal Medicine and Aged Care, Royal Brisbane and Women’s Hospital, Butterfield St, Herston, 4006 Queensland, Australia

**Keywords:** Analgesia, Epidural, Body mass index, Caesarean section, Obstetric labour, Pregnancy

## Abstract

**Background:**

Early utilisation of neuraxial anaesthesia has been recommended to reduce the need for general anaesthesia in obese parturients. The insertion and management of labour epidurals in obese women is not straight-forward. The aim of this pilot study was to compare the failure rate of extension of epidural analgesia for emergency caesarean section, in pregnant women with a body mass index (BMI) ≥ 40 kg/m^2^, to those with a BMI < 30 kg/m^2^. The results will be used to calculate the sample size of a planned prospective study.

**Methods:**

In this retrospective, (1:1) case–control pilot study, obese subjects and control subjects were selected from the obstetric database, if they delivered between January 2007 and December 2011. All subjects used epidural analgesia during labour and subsequently required anaesthesia for Category 1 or 2 Caesarean Section. Data was extracted from the patient medical record. Failure to extend was analysed using liberal and restrictive definitions. Chi-square or Fisher’s exact tests were used to detect differences between groups. Multiple logistic regression was used to examine variables predictive of extension failure.

**Results:**

There were 63 subjects in each group. The mean BMI of the obese group was 45.4 (5.8) kg/m^2^ and 23.9 (3.0) kg/m^2^ in the control group. The odds ratio for failure to extend the existing epidural blockade (liberal definition) was 2.48 (95 % CI:1.02 – 6.03) for the obese group compared with the control group (adjusted for age, parity and gestation). Using the restrictive definition, the odds ratio for failure in the obese group was 6.78 (95 % CI:1.43 – 32.2). The combination of respiratory co-morbidity and gestational diabetes significantly predicted extension failure. Surgical time and epidural complications on labour ward were significantly greater in the obese group.

**Conclusions:**

In this small retrospective cohort, patients with a BMI ≥ 40 kg/m^2^ were significantly more likely to fail epidural extension for caesarean section. The presence of respiratory co-morbidity and gestational diabetes were significant predictors of extension failure; their clinical relevance requires further evaluation.

## Background

Early utilisation of neuraxial analgesia in labour has been recommended, to avoid the risks of general anaesthesia in obese parturients if anaesthesia is required [1–3]. Early epidural catheter insertion also avoids technical difficulties later in labour when uterine contractions occur more frequently. Current guidelines recommend antenatal anaesthetic consultation for parturients with a body mass index (BMI) ≥ 40 kg/m^2,^ and consideration of early epidural analgesia [3–5]. This recommendation is based on the increased incidence of emergency caesarean delivery, instrumental delivery and macrosomic neonates in the obese population, and the predicted risks and difficulty in providing anaesthetic care [6]. While the presence of a labour epidural allows for extension to surgical anaesthesia if necessary, the insertion and management of labour epidurals in obese women is not always straight-forward [7, 8].

Problems concerning labour epidurals in obese patients include difficulty of insertion due to poorly defined landmarks, displacement of epidural catheters after insertion, and higher resite rates [6]. Dresner et al. demonstrated that as the patient BMI increases, midwife and patient dissatisfaction with the function of the epidural increases [6].

Extension of a labour epidural to achieve surgical anaesthesia utilises a large volume of a high concentration local anaesthetic solution. When extending a labour epidural in a general obstetric population, several predictors of failure have been identified. The number of clinician performed boluses during labour has consistently shown to be predictive [9–13]. A meta-analysis by Hillyard et al. suggested that the selection of local anaesthetic is important with lignocaine and adrenaline providing a faster onset neuraxial block [14]. The patient’s BMI was shown to be predictive in one study [12] but Halpern et al. found that those with a BMI >35 kg/m^2^ were not more likely to fail conversion to surgical anaesthesia [11]. The study of Halpern et al., examined 501 women, with a mean BMI of 29 ± 6.8 kg/m^2^, meaning few women with a BMI >40 kg/m^2^ would have been included. They suggested that closer attention paid to failing epidurals may account for their negative finding. Women with a BMI >40 kg/m^2^ are frequently underrepresented in published cohort studies. For instance, of Bamgade’s cohort of 1477 women undergoing caesarean section, 107 women had a BMI >40 kg/m^2^ and only eight of these had epidural extension as their primary technique [15]. There are no prospective studies specifically examining the effectiveness of extension of labour analgesia to surgical anaesthesia, in patients with a BMI ≥ 40 kg/m^2^.

The non-specific wording of guidelines suggesting early epidural analgesia for obese parturients, leaves these guidelines open to interpretation. The recommendation to request an early epidural, in order to avoid general anaesthesia, would be justifiable, if the failure rate of epidural extension in this population were known. This retrospective (1:1) case control (pilot) study aims to compare the failure rate in extending labour analgesia for caesarean section in patients with a BMI ≥ 40 kg/m^2^, compared with patients with a BMI < 30 kg/m^2^. The results will guide sample sizes for future studies, with the ultimate goal of clarifying antenatal advice given by anaesthetists to pregnant women with a BMI ≥ 40 kg/m^2^.

## Methods

Ethics approval was obtained through the Royal Brisbane and Women’s Hospital (RBWH) Human Research Ethics Committee (Reference HREC12QRBW237). The RBWH is a tertiary referral centre with approximately 4,500 deliveries per year. The anaesthetic department provides a twenty-four-hour epidural service with the majority of after-hours work performed by registrars. The overall epidural rate in 2012 was 34 %. Epidural analgesia with a patient-controlled bolus is prescribed, with a continuous background infusion via multi-orifice catheters. Bupivacaine 0.1 % and levobupivacaine 0.0625 % are the local anaesthetics used, in combination with fentanyl 2 mcg/mL. There is no set institutional protocol for anaesthetic management when a patient presents for caesarean section with an epidural in-situ. Usual practice is to assess the quality of the labour epidural and attempt to extend this analgesia if the assessment is reassuring. Our institution has three dedicated obstetric anaesthetists with the majority of consultants undertaking mixed practice. Junior staff include registrars in years two-to-four of a five year training program.

Subjects in the obese group were selected according to their BMI, calculated from the measured height and weight at the 13 week antenatal appointment. Patients with a booking-in BMI ≥ 40 kg/m^2^ between January 2007 and December 2011 were identified using the hospital Obstetric Database. This time interval was selected because after this period, ultrasound-assisted techniques became more commonly used in obese parturients. Study subjects (Group O) were selected if they used labour epidural analgesia and subsequently required Category 1 or 2 caesarean section. The RANZCOG classification of urgency for caesarean section is used by our institution [16]. Category 1 caesarean section is defined as “urgent threat to the life of a woman or fetus”. Category 2 caesarean section is defined as “maternal or fetal compromise but not immediately life threatening” [16]. Subjects in the control group were selected from the same Obstetric Database. They had a BMI < 30 kg/m^2^ documented at their 13 week antenatal appointment and also delivered between January 2007 and December 2011. A database spreadsheet of subjects with a BMI < 30 kg/m^2^ who used labour epidural analgesia and delivered by category 1 or 2 caesarean section, was used to identify controls. The subject BMI was not included on the spreadsheet and controls were selected if they delivered within the same month and year as their obese counterpart. This approach was used to avoid temporal changes in staff and management practice.

Data were extracted from the patient medical record by a consultant obstetric anaesthetist. The primary outcome measure was failure of the labour epidural to be used as the sole anaesthetic technique for caesarean section. Failure was defined as:

1. use of an alternative neuraxial technique
2. general anaesthesia was administered:
  - as a pre-operative decision, before skin incision
  - as an intra-operative decision, after skin incision

This liberal definition of extension failure has been used previously [17]. Regional anaesthesia (RA) was defined as epidural extension or a new neuraxial technique being successfully used for the duration of surgery. General anaesthesia (GA) was defined as the administration of GA pre-operatively or intra-operatively, defined above. A conversion to GA was considered to have occurred in any patient who had an epidural in-situ (all our patients) and subsequently utilised GA, consistent with the audit definition of the Royal College of Anaesthetists [18]. The data was also analysed according to a more restrictive definition of failure, including only those subjects whose anaesthetic was commenced using epidural extension.

The pregnancy health record provided information on age, parity, BMI (documented at the thirteen week antenatal appointment), previous caesarean section, co-morbidities, and pregnancy related complications. The epidural insertion details were obtained from the epidural audit document. A pre-operative complication was recorded if one or more of the following were identified: ineffective analgesia, catheterisation of an epidural vein, re-site required or accidental dural puncture. The number of anaesthetist interventions was not reliably documented, however inadequate analgesia documented by midwifery staff was considered a surrogate marker of the requirement for anaesthetist attendance. The category of urgency of the caesarean section and indication for delivery were obtained from the theatre record or labour ward notes. The indication was classified according to whether it was primarily for maternal or foetal reasons (maternal reasons: pre-existing condition, pregnancy-related condition, complication of labour or delivery; foetal reasons: CTG abnormal, abnormal presentation). Failure to progress, a common indication for caesarean section was considered a maternal indication, unless there was evidence of foetal compromise, in which case it was classified as a foetal indication – CTG abnormal.

The anaesthetic record was used to determine: the method of anaesthesia, documented airway concerns (known history of Cormack and Lehane class 3 or 4 or “potential difficult airway” documented), epidural medications utilised (if extension was attempted), supplementation of RA, conversion to GA and whether this occurred pre-operatively or intra-operatively, and the seniority of anaesthetist. Senior anaesthetists included Senior Registrars (year 5 of a five year program) and Consultants. The duration of the surgical procedure was obtained from the theatre record’s compulsory fields of “OT in” (time of entry to theatre) and “OT out” (patient exited theatre). In our institution the regional anaesthetic is performed/extended in the induction room, except for Category 1 cases which are transferred directly on to the operating theatre table where epidural extension commences.

The primary outcome variable was binary and summarised using frequencies and percentages. Chi-square or Fisher’s exact tests were used to detect differences between groups. Mann–Whitney U test was used for continuous variables. The odds ratio was adjusted using the simple baseline variables of age, parity and gestation. To identify potential predictors of extension failure, explanatory variables were screened for inclusion in multiple regression modelling. Forward and backward stepwise selection procedures were used. The explanatory variables considered for inclusion were those that could be identified at an antenatal anaesthetic consultation: BMI (documented at the thirteen week antenatal appointment), history of previous caesarean section, a documented airway concern, suspected or documented obstructive sleep apnoea, gestational diabetes, gestational hypertension and co-morbidities (respiratory disease, cardiovascular disease, and mental illness). A p-value < 0.05 was considered to signify statistical significance.

## Results

**Fig. 1:**
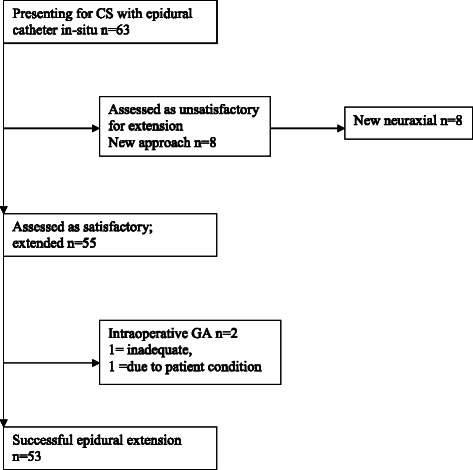
Control Group. Flow chart of anaesthetic management in 63 women presenting for emergency caesarean section with an epidural catheter in situ, body mass index < 30 kg/m^2^. CS = caesarean section GA = general anaesthesia

**Fig. 2:**
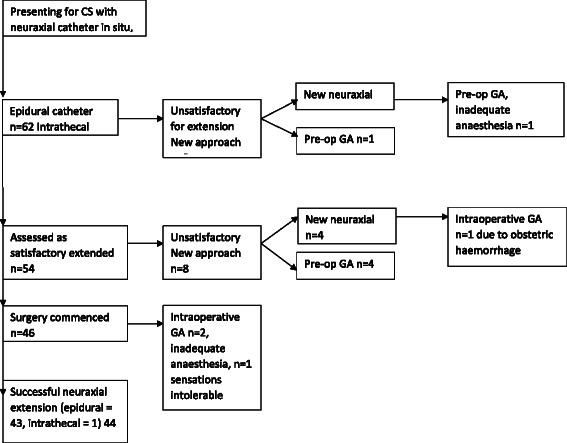
Obese Group. Flow chart of anaesthetic management of 63 women presenting for emergency caesarean section with a neuraxial catheter in-situ, body mass index ≥ 40 kg/m^2^. CS = Caesarean section; Pre-op = preoperative; GA = general anaesthesia

A total of 63 subjects were identified for the obese group; data was collected for 63 subjects in the control group who delivered within the same month. The mean (SD) BMI of 23.9 (3.0) kg/m^2^ in the control group and 45.4 (5.8) kg/m^2^ in the obese group. Ultrasound localisation was not utilised in any patient. Figures 1 and 2 describe the management of subjects in the obese group and control group when they presented for caesarean section with a neuraxial catheter in situ. The baseline variables and co-morbidities are shown in Table 1. Of the twenty-six obese subjects with a respiratory co-morbidity, twenty-two had a self-reported history of asthma.

**Table 1:** Demographic and co-morbidity data of 126 women delivering by emergency caesarean section 2007–2011 at the Royal Brisbane and Women’s Hospital

Variable	Group C	Group O	*p value*
	*n* = 63	*n* = 63	
Age (years) mean (SD)	29.7 (5.0)	30.6 (5.6)	0.33
Nulliparous n (%)	49 (77.8)	53 (84.1)	0.36
Gestation > 39 weeks n (%)	51 (81.0)	42 (66.7)	0.17
VBAC^a^ n (%)	6 (9.5)	2 (3.2)	0.27
Comorbidities n (%)			
Respiratory^b^	9 (14.3)	26 (41.3)	0.001
Mental health^c^	15 (23.8)	12 (19.0)	0.52
Documented airway concerns	0 (0)	2 (3.2)	0.5
Sleep apnoea (Suspected/known)	0 (0)	2 (3.2)	0.5
Gestational hypertension^d^	5 (7.9)	21 (33.3)	<0.001
Gestational diabetes^e^	4 (6.3)	13 (20.6)	0.02

Data is presented as mean (SD) or number (%)

^a^ VBAC Vaginal birth after caesarean section

^b^Respiratory: Smoking, asthma or current infection

^c^Mental Health: Anxiety or Depression

^d^ Criteria according to International Society for the Study of Hypertension in Pregnancy 2000

^e^Criteria according to Australasian Diabetes in Pregnancy Society 1998

Table 2 shows the primary and secondary outcome measures.

**Table 2:** Labour epidural and anaesthetic details for 126 women delivering by Caesarean Section, 2007–2011 at the Royal Brisbane and Women’s Hospital

Variable	Group C	Group O	*p value*
	*n* = 63	*n* = 63	
Insertion of labour epidural			
Senior anaesthetist n (%)	7 (11.3)	13 (21.0)	0.14
Number of attempts ≥ 2 n (%)	18 (28.6)	34 (59.6)	0.001
Depth to space cm (median) (IQR^a^)	5 (4.5-6.0)	8.0 (7.0-8.0)	<0.001
Length of catheter in epidural space cm (median) IQR	4.5 (4.0-5.0)	5.0 (4.0-5.0)	0.29
Complication n (%)	8 (12.7)	22 (34.9)	0.003
resite (n)	3	10	
ineffective(n)	2	9	
intravascular (n)	3	3	
dural puncture (n)	-	1	
Anaesthesia for caesarean section			
Extension failure n (%) (liberal definition)	10 (15.9)	20 (31.7)	0.04
Category 1 CS n (%)	5 (7.9)	9 (14.3)	0.26
Indication: Failure to progress n (%)	43 (68.3)	44 (71.0)	0.74
Senior anaesthetist n (%)	7 (11.1)	24 (40.0)	<0.001
Caesarean section surgical time (min) median (IQR)	66 (17)	79 (26.0)	<0.001

Data is presented as number (%) or median (interquartile range)

^a^IQR = interquartile range

Using the liberal definition of failure, the rate was 15.9 % in the control group and 31.7 % in the obese group. The unadjusted odds ratio for failure to extend was 2.47 (95 % CI: 1.04 – 5.82) for the obese group compared with the control group. When adjusted for age, parity and gestation, the odds ratio remained significant (2.48, 95 % CI:1.02 – 6.03).

Table 3 shows three logistic regression models of factors significantly associated with extension failure. A parsimonious approach was applied to obtain the most clinically interpretable model; using the least number of predictors to achieve the highest pseudo-R2 value. A higher pseudo-R2 indicates a better model fit. Only a small improvement in pseudo-R2 is obtained by adding BMI as a predictor. The combination of respiratory disease and gestational diabetes were the two factors that together were most predictive of extension failure. Those subjects who failed extension were also more likely to have a senior anaesthetist present at caesarean section (X^2^ = 4.43, df = 1, p = 0.04).

**Table 3:** Three logistic regression models of factors predicting extension failure. The third model, is the simplest and most predictive

Variable	Odds ratio (OR)	95 % CI of OR	Wald’s X^2^	*p-value*	^a^Pseudo-R^2^
Model 1					
Respiratory co-morbidity	3.13	1.32 - 7.42	6.66	0.01	0.048
Model 2					
Respiratory co-morbidity	2.74	1.09 - 6.87	4.62	0.03	0.078
^b^Gestational diabetes	2.34	0.77 - 7.37	2.25	0.13	
^c^BMI	1.67	0.66 - 4.24	1.17	0.28	
Model 3					
Respiratory co-morbidity	3.19	1.32 - 7.70	6.66	0.01	0.069
Gestational diabetes	2.72	0.90 - 8.27	3.13	0.08	

^a^Pseudo-R2: a higher value indicates a better model fit

^b^Criteria according to Australasian Diabetes in Pregnancy Society

^c^BMI = body mass index

One subject in Group O received an intrathecal catheter after inadvertent dural puncture; this was managed by intermittent anaesthetist-administered intrathecal bolus and successfully extended for caesarean section. (This was classified as a failed extension, as it was a complication of the primary technique.) The remainder received epidurals (no spinal component) and patient-controlled epidural analgesia with a background infusion. The most common indication for caesarean section was failure to progress (Table 2). Foetal heart rate abnormalities accounted for the remaining cases, other than one abnormal foetal presentation in the control group.

The conversion rate from RA to GA was 14.3 % (95 % CI: 5.66-22.94) in the obese group and 3.2 % (95 % CI: −1.15-7.55) in the control group (X^2^ = 4.88, df = 1, p = 0.03). In six of the nine GAs in the obese group, the decision for GA was made preoperatively and all of these occurred after failure of regional anaesthesia. The remainder occurred intra-operatively. Of the 97 subjects with successfully extended epidurals, three subjects in the obese group and six subjects in the control group received supplemental analgesia or sedation. Of the 11 new neuraxial blocks performed in the obese group, six were performed by senior anaesthetists compared with three by junior anaesthetists (two had missing data).

The mean number of attempts at epidural insertion and the occurrence of a pre-operative complication were both greater in the obese group. The odds of epidural insertion attempts of ≥ 2 was 3.10 times higher (95 % CI: 1.40 – 6.8) in the obese group compared with the control group. The odds ratio for a pre-operative complication was 2.83 (95 % CI: 1.1-7.27) in the obese group compared with the control group.

Table 4 shows the composition of epidural medications used, for those subjects whose epidural was extended.

**Table 4:** Composition and volume of medications used for epidural extension

Medication Details	Group C (*n* = 56)	Group O (*n* = 50)	*p-value*
Local Anaesthetic type n (%)^a^			
2 % Lidoocaine & adrenaline	30 (53.6)	34 (68)	0.13
Other^b^	25 (46.4)	16 (32.0)	
Local Anaesthetic volume mLs median (IQR)	17.5 (7.8)	18 (5.0)	0.23
Additive n (%)			
Additive^c^	41 (73.2)	41 (82.0)	0.36
No additive	15 (26.8)	9 (18.0)	

Data is presented as number (%) or median (interquartile range)

^a^ Data missing, *n* = 1

^b^Ropivacaine 0.75 % n = 36; bupivacaine 0.5 % *n* = 1; mixture n = 4

^c^Fentanyl n = 77; fentanyl and clonidine *n* = 3; bicarbonate: n = 2

Using a more restrictive definition of extension failure revealed similar results. This definition included only those whose anaesthetic commenced with extension of their epidural. Of the 55 control subjects who had an epidural extension, only two (3.6 %), required use of another technique. In the obese group, eleven of 54 (20.4 %) subjects having epidural extension required another technique. Those in the obese group were more likely to fail extension and the difference was significant (X2 = 7.26, df = 1, p = 0.007). The odds ratio for failure to extend (using the more restrictive definition) in the obese group was 6.78 (95 % CI:1.43 – 32.2).

## Discussion

Extension of epidural analgesia for emergency caesarean section was significantly more likely to fail in obese subjects than in control subjects. The combined presence of a respiratory co-morbidity with gestational diabetes most significantly predicted extension failure. Asthma was the most common respiratory co-morbidity in this population.

The association between asthma and obesity is complex [19]. A recent meta-analysis suggests that there is a 50 % increase in asthma in overweight or obese individuals [20]. In addition, there is evidence that surgical weight management strategies can reduce the severity of asthma in obese patients [21]. Respiratory disease may reduce the ability of a woman to cope with caesarean section under regional anaesthesia, making conversion to GA more likely. Underling mechanisms include exacerbation of dyspnoea by further reduction of functional residual capacity by the supine position, intercostal muscle paralysis due to regional anaesthesia and extreme surgical retraction. It is also possible that the presence of a respiratory comorbidity alters decision-making by anaesthetists, making them more likely to institute a new neuraxial technique rather than extending the existing epidural in an effort to avoid general anaesthesia and intubation.

The incidence of respiratory disease in the obese group is particularly high. With retrospective data collection it was not possible to collect information regarding the diagnosis and management of “self-reported” asthma. Without formal spirometry results on each patient, it is not possible to assess which of these patients may have shortness of breath related to their obesity, rather than asthma.

Obese parturients are known to have a higher rate of gestational diabetes than the non-obese parturients. A meta-analysis by Chu et al. estimated that the odds ratio of developing gestational diabetes was 3.56 for an obese woman compared to a non-obese woman and that the odds ratio rises with increasing BMI [22]. While its influence on extension failure is unclear, for our small, retrospectively examined cohort, the combination of gestational diabetes with respiratory co-morbidity strongly predicted those subjects whose pre-existing labour epidural was not used for caesarean section anaesthesia. This potential relationship requires further analysis in a prospective study.

Emergency surgery involving parturients with a BMI ≥ 40 kg/m^2^ is considered high risk anaesthesia. The attendance of senior anaesthetists at these cases is expected and not surprising. Failure to extend the labour epidural was more common in the presence of a senior anaesthetist. Senior clinicians may have a higher degree of comfort in performing de novo neuraxial anaesthesia in obese women and therefore a lower threshold for removing the labour epidural, rather than risk failure of epidural extension. Although institutional rates of epidural extension failure vary [17, 23, 24], it is accepted that there is a failure rate and it can be argued that important time may be saved, by proceeding directly to a new neuraxial technique. Similarly, a senior clinician may be more confident in providing general anaesthesia to these high risk patients if they feel it is warranted. There were more Category 1 caesarean sections in the obese group (although not statistically significant) and this may also have hindered the extension of labour epidurals. If these factors all influence whether or not a pre-existing epidural is extended, it calls into question antenatal recommendations made to obese pregnant women, to have an early epidural on the basis that it will be used as their primary anaesthetic technique if caesarean section becomes necessary.

The extension failure rate (using the liberal definition) of 15.9 % in the control group is at the higher end of the range reported in the literature. Eight new neuraxial blocks used in the control group contributed significantly to this failure rate. Using the same definition of failure, Pan et al. [17] reported a failure rate of 7.1 % in 4190 patients utilising labour epidural. The selection and volume of local anaesthetic used in both of our groups were consistent with currently accepted practice and like the study of Pan et al. [17] the large majority were inserted on labour ward by junior staff. The mean depth to the epidural space was significantly greater in the obese group, consistent with previous reports. In a 2011 review, Mace et al. [8] suggested leaving a catheter length of 5–6 cm in the epidural space of obese women to minimise dislodgement. The mean catheter length inserted into the epidural space in the obese group was consistent with this (Table 2), although other authors have suggested up to 7 cm is required [25]. Leaving more catheter length within the epidural space may reduce dislodgement, but increases the likelihood of intravascular catheterisation or unilateral analgesia [26].

Despite the high rate of “failure to extend”, the conversion rate to GA in the control group was low and less than the 5 % suggested as an audit target by the Royal College of Anaesthetists [18]. In comparison the conversion rate of 14.3 % in the obese group (using the liberal definition) was outside that target. The higher rate of general anaesthesia in the obese group is consistent with results published from the United Kingdom. The United Kingdom Obstetric Surveillance System, found that women with extreme obesity (BMI ≥ 50 kg/m^2^) were six times more likely to have a general anaesthetic than the less obese comparison group [27].

The management of epidurals in the obese group on labour ward was more complicated and this has been observed previously [28, 29]. The most common difficulty was inadequate analgesia and the necessity to resite the epidural. Inadequate analgesia is known to be associated with failure to extend a labour epidural [13, 30].

Using the more liberal definition of extension failure, this data indicates the probability of extension failure in controls is 0.159 (there were 10 out of 63 extension failures in that group). With the odds ratio of extension failure in obese subjects found to be 2.48, a prospective study would require 110 subjects in each group, to demonstrate a clinically significant difference between groups, with power of 0.8 and significance level of 0.05. It is proposed that a clinically significant failure rate in obese patients would be twice that observed in controls.

The retrospective methodology used in this study limits the impact of the results. Potential predictors, such as number of clinician boluses on labour ward were represented by a surrogate endpoint. Other relevant data, which was not available included information on the use, timing and location of a test dose, the decision-to-delivery interval, and details of asthma diagnosis and treatment. Lack of standardised practice in our institution is a limitation, however audit of local practice reveals homogenous practice in terms of volume and selection of local anaesthetic used for epidural extension. The precision of results in this study would have been improved by 1:2 matching and this will be used in the prospective study. As a pilot study these results provide important information regarding data collection and sample size, which will be applied in a prospective study.

## Conclusion

The results of this pilot study should be interpreted with caution, in view of the small sample size and retrospective methodology. Despite this, the results support the observation that a BMI ≥ 40 kg/m^2^ may be associated with greater difficulty extending labour analgesia, than in those patients BMI < 30 kg/m^2^. This is clinically relevant, given the recommendations made to obese parturients in the antenatal period. Obese women with the combination of co-existing respiratory disease and gestational diabetes may be particularly at risk of extension failure. A prospective study will be required to substantiate these findings.

## Abbreviations

BMI: Body mass index
RA: Regional anaesthesia
GA: General anaesthesia

## References

[1] Soens MA, Birnbach DJ, Ranasinghe JS, van Zundert A (2008). Obstetric anesthesia for the obese and morbidly obese patient: an ounce of prevention is worth more than a pound of treatment. Acta Anaesthesiol Scand.

[2] Practice Guidelines for Obstetric Anesthesia: An Updated Report by the American Society of Anesthesiologists Task Force on Obstetric Anesthesia. United States of America: American Society of Anesthesiologists; 2007. 10.1097/01.anes.0000264744.63275.10 17413923

[3] Queensland Maternity and Neonatal Guideline: Obesity. Queensland State Government, Queensland, Australia. 2010, Accessed January 2015. www.health.qld.gov.au/qcg.

[4] Joint Guideline for the Management of Women with Obesity in Pregnancy. Centre for Maternal and Child Enquiries; Royal College of Obstetricians and Gynaecologists, United Kingdom. 2010. https://www.rcog.org.uk/en/guidelines-research-services/guidelines/management-of-women-with-obesity-in-pregnancy/. Accessed June 2015.

[5] Davies GAL, Maxwell C, McLeod L, Gagnon R, Basso M, Bos H (2010). SOGC Clinical Practice Guidelines: Obesity in pregnancy. No. 239, February 2010. Int J Gynaecol Obstet.

[6] Dresner M, Brocklesby J, Bamber J (2006). Audit of the influence of body mass index on the performance of epidural analgesia in labour and the subsequent mode of delivery. BJOG.

[7] Saravanakumar K, Rao SG, Cooper GM (2006). The challenges of obesity and obstetric anaesthesia. Curr Opin Obstet Gynecol.

[8] Mace HS, Paech MJ, McDonnell NJ (2011). Obesity and obstetric anaesthesia. Anaesth Intensive Care.

[9] Bauer ME, Kountanis JA, Tsen LC, Greenfield ML, Mhyre JM (2012). Risk factors for failed conversion of labor epidural analgesia to cesarean delivery anesthesia: a systematic review and meta-analysis of observational trials. Int J Obstet Anesth.

[10] Campbell DC, Tran T (2009). Conversion of epidural labour analgesia to epidural anesthesia for intrapartum Cesarean delivery. Can J Anaesth.

[11] Halpern SH, Soliman A, Yee J, Angle P, Ioscovich A (2009). Conversion of epidural labour analgesia to anaesthesia for Caesarean section: a prospective study of the incidence and determinants of failure. Br J Anaesth.

[12] Orbach-Zinger S, Friedman L, Avramovich A, Ilgiaeva N, Orvieto R, Sulkes J (2006). Risk factors for failure to extend labor epidural analgesia to epidural anesthesia for Cesarean section. Acta Anaesthesiol Scand.

[13] Lee S, Lew E, Lim Y, Sia AT (2009). Failure of augmentation of labor epidural analgesia for intrapartum cesarean delivery: a retrospective review. Anesth Analg.

[14] Hillyard SG, Bate TE, Corcoran TB, Paech MJ, O'Sullivan G (2011). Extending epidural analgesia for emergency Caesarean section: a meta-analysis. Br J Anaesth.

[15] Bamgbade O, Khalaf W, Ajai O, Sharma R, Chidambaram V, Madhavan G (2009). Obstetric anaesthesia outcome in obese and non-obese parturients undergoing caesarean delivery: an observational study. Int J Obstet Anesth.

[16] C-Obs 14 Categorisation of urgency for caesarean section. The Royal Australian and New Zealand College of Obstetricians and Gynaecologists Australia. 2012. https://www.ranzcog.edu.au/college-statements-guidelines.html. Accessed June 2015

[17] Pan PH, Bogard TD, Owen MD (2004). Incidence and characteristics of failures in obstetric neuraxial analgesia and anesthesia: a retrospective analysis of 19,259 deliveries. Int J Obstet Anesth.

[18] Raising the standard (2012). A compendium of audit receipes.

[19] Beuther DA, Weiss ST, Sutherland ER (2006). Obesity and asthma. Am J Respir Crit Care Med.

[20] Beuther DA, Sutherland ER (2007). Overweight, obesity, and incident asthma: a meta-analysis of prospective epidemiologic studies. Am J Respir Crit Care Med.

[21] O'Brien PE, Dixon JB, Brown W, Schachter LM, Chapman L, Burn AJ (2002). The laparoscopic adjustable gastric band (Lap-Band®): a prospective study of medium-term effects on weight, health and quality of life. Obes Surg.

[22] Chu SY, Callaghan WM, Kim SY, Schmid CH, Lau J, England LJ (2007). Maternal obesity and risk of gestational diabetes mellitus. Diabetes Care.

[23] Garry M, Davies S (2002). Failure of regional blockade for caesarean section. Int J Obstet Anesth.

[24] Kinsella SM (2008). A prospective audit of regional anaesthesia failure in 5080 Caesarean sections. Anaesthesia.

[25] Iwama H, Katayama T (1999). Back skin movement also causes “walking” epidural catheter. J Clin Anesth.

[26] Gorman D, Birnbach D. In: Cousins M, Phillip B, editors. Cousins and Bridenbaugh's neural blockade in clinical anesthesia and pain medicine. Fourth ed.: Lippincott Williams & Wilkins; 2009. p. 544–5.

[27] Knight M (2010). Extreme obesity in pregnancy in the United Kingdom. Obstet Gynecol.

[28] Perlow JH, Morgan MA (1994). Massive maternal obesity and perioperative cesarean morbidity. Am J Obstet Gynecol.

[29] Hood DD, Dewan DM (1993). Anesthetic and obstetric outcome in morbidly obese parturients. Anesthesiology.

[30] Orbach-Zinger S, Friedman L, Avramovich A, Ilgiaeva N, Orvieto R, Sulkes J (2006). Risk factors for failure to extend labor epidural analgesia to epidural anesthesia for Cesarean section. Acta Anaesthesiol Scand.

